# A New Operation for Paralytic Talipes Valgus and the Enunciation of a New Surgical Principle

**Published:** 1893-01

**Authors:** 


A NEW OPERATION FOR PARALYTIC TALIPES VALGUS AND THE ENUNCIATION OF A NEW SURGICAL PRINCIPLE.—Parrish (*New York Medical Journal*, October 8, 1892), says the greater number of these deformities results from polio-myelitis. The muscles of the lower limbs are most frequently affected by this disease. The muscles of the legs are more frequently involved than those of the thigh. Of the leg muscles, the anterior and posterior tibialis, the muscles of the calf and the peroneals are in the order named the most frequently affected. It is well-known that the extensor pollicis, which is a very strong muscle, and seldom affected by paralysis, might bear the burden of its weaker neighbor. After isolating both the anterior tibial and extensor pollicis muscles, the foot was placed in the position of inversion and extention, and the shorter tendon of the first sewed to the lengthened tendon of the latter. The two tendons were first sewed together above the annular ligament. Then, placing the foot in the position of extreme eversion, the author pulled upon the belly of the extensor pollicis muscle, and when the foot returned from its everted position, the arch was raised and the great toe was extended. On another subject the two tendons were sewed below the annular ligament, and the experiments were repeated with equally satisfactory results. After being convinced that the operation would be a success, it was tried on a little girl, aged 3 years and 10 months, who had had infantile paralysis when 11 months old. At the time of operation both tibial muscles of the right leg were completely paralyzed. All the other muscles which had been involved recovered.

Under chloroform an incision was made over the space between the tendons of the anterior tibial and the extensor pollicis muscle, extending from the annular ligament three inches and a half upwards. Both the tendons were found and isolated. The tendon sheaths were cut away and the foot everted, so as to shorten the tendon of the anterior tibial and lengthen that of the extensor pollicis. The opposing tendon surfaces were then freshened with a knife and sewed together with cat-gut suture for the space of an inch or more, and the wound closed. The foot was then placed in its proper position and retained so by a plaster-of-paris bandage, which was worn for a month. When the dressing was removed the wound was found perfectly healed and the foot in much better position than before. What the final outcome will be the author is, as yet, unable to say.—*University Med. Mag.*

